# A new socioeconomic status measure for vaccine research in children using individual housing data: a population-based case-control study

**DOI:** 10.1186/s12889-016-3673-x

**Published:** 2016-09-21

**Authors:** Rachel Hammer, Conrad Capili, Chung-Il Wi, Euijung Ryu, Jennifer Rand-Weaver, Young J. Juhn

**Affiliations:** 1 Tulane University School of Medicine, 1440 Canal Street, Suite 1000, New Orleans, LA 70112 USA; 2 Children’s Medical Center, 1935 Medical District Drive, Dallas, TX 75235 USA; 3 Division of Community Pediatric and Adolescent Medicine, Department of Pediatric and Adolescent Medicine, Mayo Clinic, 200 First Street SW, Rochester, MN 55905 USA; 4 Department of Health Sciences Research, Mayo Clinic, 200 First Street SW, Rochester, MN 55905 USA; 5 Rochester-Olmsted Planning Department, 2122 Campus Drive SE, Rochester, MN 55904 USA

**Keywords:** Epidemiology, Vaccination, Socioeconomic status

## Abstract

**Background:**

We recently developed HOUSES, an individual housing-based socioeconomic status (SES) measurement for health disparities research. We assessed whether HOUSES was associated with risk of pertussis and pertussis vaccine up-to-date status in children.

**Methods:**

The study utilized a previous population-based case-control study cohort assembled during the 2004–2005 pertussis outbreak. We collected data on pertussis vaccine status (up-to-date status) at the time of the index date. Using a z-score for housing value, actual square footage, and numbers of bedrooms and bathrooms, HOUSES was formulated in continuous variable and categorized into quartiles. Vaccine up-to-date status was compared among subjects with different SES as measured by HOUSES using a chi-square test and logistic regression models.

**Results:**

Of the 391 eligible pediatric subjects (median age of 13.1 years with male sex of 55 %), 363 (93 %) were successfully geocoded to formulate HOUSES index. HOUSES was not associated with the risk of pertussis (*p* = 0.82). Pertussis vaccine up-to-date statuses were 79, 86, 83, and 94 % for children in the first (the lowest SES), second, third, and fourth quartiles of HOUSES, respectively (*p* = 0.03). HOUSES as a continuous variable was associated with pertussis vaccine up-to-date status (adjusted OR: 1.15 per increment of one unit of HOUSES, 95 % CI: 1.04–1.27, *p* = 0.008).

**Conclusion:**

While HOUSES is not associated with the risk of pertussis, it predicts vaccine up-to-date status among children with different SES. HOUSES may be a useful tool for vaccine delivery research among children.

## Background

Pertussis is re-emerging as an infectious disease causing significant morbidity, through the social burden of lost workdays and school-days, and mortality, especially among infants [1]. Pertussis outbreaks are on the rise with more than 48,000 reported cases per the CDC in the United States in 2012, as compared with 27,550 in 2010. These statistics are staggering when bearing in mind the historic low of 1010 cases in 1976. It is estimated that 50 % of pertussis cases affect children under the age of 16, a population in whom the effects of the infection can be serious [1]. In 2010, there were 6257 pertussis cases reported in California, including nine infants under 2 months of age who died having not received the pertussis vaccine [2].

Known risk factors for pertussis include household exposure to susceptible individuals, close proximity to a cough under 5 ft from a carrier, being female, and being an infant [3–5]. Recently, asthma has been shown to increase the risk of pertussis. Additionally, the efficacy of the acellular pertussis vaccine has been called into question and possibly implicated as an important factor in recent outbreaks [6, 7], and notably in the 2012 outbreak in Southeast Minnesota [8], wherefrom our study cohort derives.

Socioeconomic Status (SES) is an important factor to be considered in understanding the epidemiology of all infectious diseases, including pertussis, because environment and resources impact multiple domains related to the risk of contracting and spreading an infectious disease. Indeed, the risk of acute and chronic respiratory diseases has been shown to increase among populations of lower SES, especially during childhood [9–11].

While SES is an important factor to be considered for the epidemiology of infectious diseases, it is frequently unavailable in commonly used datasets. As a result, area (census)-level SES measures are increasingly used as a proxy for individual SES [12–18]. For example, in our previous report showing the relationship between asthma and the increased risk of pertussis, only about 30 % of subjects had parental education levels available in medical records [19]. To address this concern, we developed an SES index derived from publicly available housing features including: square footage, assessed housing value, and the number of bedrooms and bathrooms in the residence where a patient lives [20]. This index, the HOUsing-based measure of SocioEconomic Status (*termed as* HOUSES), has proven its utility as a measure for SES in predicting various health outcomes including: low birth weight, obesity, household smoking exposure, invasive pneumococcal diseases, asthma control status, and general health status in children [20–23], as well as the risk and mortality of rheumatoid arthritis [24] and post-myocardial infarction mortality in adults [25].

Due to the large number of subjects with missing SES data in our previous study, we did not adequately address the role of SES in the risk of pertussis and up-to-date pertussis vaccination status. Thus, we sought to assess the effect of HOUSES on the risk of pertussis and up-to-date pertussis vaccination status. We hypothesized that HOUSES might impact the risk of pertussis through differential pertussis vaccine up-to-date status (up-take rate) among children with different SES measured by HOUSES.

## Methods

The study was approved by both the Institutional Review Boards at Mayo Clinic and Olmsted Medical Center.

### Study population and setting

Rochester, Minnesota, is centrally located in Olmsted County. During the study period, characteristics of the City of Rochester and Olmsted County populations were similar to those of the U.S. Caucasian population, with the exception of a higher proportion of the working population employed in the health care industry [26]. Health care in Olmsted County, Minnesota, is virtually self-contained within the community. Uniquely, when patients first register care with any health care provider in the community, they are asked to authorize the use of their medical records for research. Those who grant such authorization (and 95 % do), are assigned a unique identifier with the Rochester Epidemiology Project (REP), a program which has been continuously funded by the NIH since 1966. All clinical diagnoses and information from every episode of care are contained within detailed patient-based medical records. This unique longitudinal population-based resource has been the source of over 2000 publications on the epidemiology of disease.

### Study design

The study was designed as a population-based case-control study. We utilized a previous population-based study cohort, which included 164 pertussis cases and 328 age- and gender-matched controls among Olmsted County, Minnesota, residents during the 2004–2005 pertussis outbreak. In this study, we only evaluated the pediatric cohort, defined as individuals under 18 years of age at the time of the index date.

### Study subjects: cases and controls

Details about the study cohort have been previously described [19]. Briefly, a case was defined as isolation of *Bordetella pertussis* by PCR from upper respiratory tract or serologic testing among Olmsted County residents between January 1, 2004, and December 31, 2005. An age- and gender-matched control group was randomly selected from a list of Olmsted County residents with negative PCR results within a month of the index date of a pertussis case. Two controls were matched for each case with regard to gender and birthday (within 1 month for patients younger than 2 years of age, within 3 months for patients between ages 2 and 5 years, within 6 months for patients between ages 6 and 12 years, and within 1 year for patients older than 12 years). Additional criteria for controls required that participants be residents of Olmsted County for least 1 year prior to the study period; the same exclusion criteria for the cases were also applied.

### Dependent variable

We collected pertussis vaccination status at the time of the index date of pertussis for both cases and controls from the medical record (DPT, DTaP, and Tdap). We defined pertussis vaccine as up to date if subjects completed all age-appropriate pertussis vaccinations as recommended by the Advisory Committee on Immunization Practices (ACIP). In general, up-to-date vaccine status meant one through five immunizations appropriate for age in the DTap (or DTP before 1997) series for children younger than 6 years of age. Since routine use of Tdap for adolescents aged ≥11 years old was recommended by ACIP in March, 2006, we did not consider Tdap for up to date vaccine status in the present study [27].

### Socioeconomic status measurement (independent variable)

We utilized two individual-level SES variables including maternal and paternal education level and an individual-housing based SES measure (HOUSES). For both cases and controls, maternal and paternal education levels and home addresses were abstracted from the entire medical record. HOUSES is a composite index derived from housing features of real property data and home address information as charted in the patient’s medical record at the time of their pertussis event. Development and initial testing of the index were completed in both Olmsted County, Minnesota, and Jackson County, Missouri. Results from that study have been reported in previous publications [20–23]. Briefly, in calculating HOUSES, each subject’s address at the index date of their pertussis event was geocoded. Geocoding allowed us to match study subject address to real property data. Once geocoded, we applied principal components factor analysis on the basis of real property data features of housing and neighborhood SES items. Factor analysis results were pared down to four real property feature variables, including market housing value, square footage of the housing unit, the number of bedrooms, and number of bathrooms. We then formulated a standardized HOUSES index score by transforming the four variables to z-scores (i.e., standardized the index to allow for comparisons across different study settings) and summing the z-scores to the HOUSES index. Essentially, a higher HOUSES index score represents a higher SES.

### Other pertinent variables

Other information abstracted from the medical records included sociodemographic variables: age, gender, ethnicity, parental education levels, birth weight, gestational age, comorbid conditions, and smoking status at the time of index date (either active or passive exposure to tobacco smoke).

### Data analysis

HOUSES was analyzed as both a continuous variable and as categorical variables (quartiles). We compared the frequency of subjects with different SES measured by HOUSES in quartiles between cases and controls to determine relationship between HOUSES and the risk of pertussis. Similarly, we determined the relationship between HOUSES and pertussis vaccine up to date status. Adjusting for age and sex, data were fitted to logistic regression models to determine the association of HOUSES with the risk of pertussis and pertussis vaccine up-to-date status by calculating odds ratios and their corresponding 95 % confidence intervals (CI). Similar analysis was performed for association of maternal and paternal education level. The weighted kappa index for the agreement between HOUSES and maternal and paternal education levels in quartiles was also calculated.

## Results

### Study subjects

The characteristics of the subjects included in the original case-control study are summarized in Table 1. Of the 492 original cohort, 391 (79.5 %) were pediatric subjects with median age of 13.1 years and male sex of 55 %. Among 391 eligible children, 363 (93 %) were successfully geocoded to formulate HOUSES index (median: −0.42, interquartile range: −2.12–2.24), and 373 (95 %) had pertussis vaccine status available (96 % in cases vs. 87 % in controls; *p* = 0.10). Three hundred forty-seven (89 %) subjects had both HOUSES data and pertussis vaccine status available for this study. The weighted kappa index for the agreement between HOUSES and maternal and paternal education levels in quartiles was 0.30 (95 % CI: 0.22–0.37) and 0.38 (95 % CI: 0.25–0.51). Maternal and paternal education levels were only available for 133 subjects (34 %) and 99 (25 %), respectively.

**Table 1:** Characteristics of pertussis cases and controls (*N* = 391)

	Controls (*n* = 261)	Cases (*n* = 130)	*P* value*
Age at case index date, years			0.90
Median (IQR)	13.1 (10.7–15.1)	13.1 (10.9–14.9)	
Mean (SD)	12.2 (3.9)	12.2 (3.8)	
Sex, no. (%)			1.00
Male	143 (54.7)	71 (54.6)	
Female	118 (45.3)	59 (45.4)	
Ethnicity, no. (%)			0.76
Hispanic/Latino	3 (1.1)	1 (0.8)	
American Indian/Alaska Native	3 (1.1)	0	
Asian	0	2 (1.6)	
Native Hawaiian/Pacific Islander	4 (1.5)	0	
White	200 (76.7)	105 (80.4)	
Unknown	51 (19.6)	22 (17.2)	
Ethnicity, no. (%)			0.51
Nonwhites	10 (3.9)	3 (2.3)	
Whites	200 (76.7)	105 (80.8)	
Unknown	51 (19.4)	22 (16.9)	
Maternal educational level, no. (%)			0.40
High school or less	16 (6.2)	10 (7.7)	
Some college	29 (11.1)	19 (14.6)	
College graduate	23 (8.8)	13 (10)	
Graduate degree or greater	18 (6.9)	5 (3.9)	
Unknown	175 (67.0)	83 (63.8)	
Paternal educational level, no. (%)			0.19
High school or less	8 (3.1)	7 (5.4)	
Some college	20 (7.7)	13 (10.0)	
College graduate	17 (6.5)	2 (1.5)	
Graduate degree or greater	23 (8.8)	9 (6.9)	
Unknown	193 (74.0)	99 (76.2)	
HOUSES index in z-score			0.82
Median (IQR)	−0.30 (−2.28–2.25)	−0.43 (−2.00–2.36)	
Mean (SD)	0.05 (3.68)	0.09 (3.29)	
HOUSES index in quartiles			0.91
First (lowest SES)	63/252 (25.0)	24/111 (21.6)	
Second	60/252 (23.8)	29/111 (26.1)	
Third	61/252 (24.2)	28/111 (25.2)	
Fourth (highest SES)	68/252 (27.0)	30/111 (27.0)	
Up-to-date status of pertussis vaccination, no. (%)			0.10
Not up to date	32 (12.3)	23 (17.7)	
Up to date	216 (82.8)	102 (78.5)	
Unknown	13 (4.9)	5 (3.8)	
Smoking status, no. (%)			0.84
Non-smoking	75 (28.7)	39 (30)	
Smoking	34 (13.0)	18 (13.8)	
Unknown	152 (58.3)	73 (56.2)	
Comorbid conditions at index date, no. (%)^a^			1.00
Yes	6 (2.3)	3 (2.3)	
No	255 (97.7)	127 (97.7)	

*By conditional logistic regression

^a^Comorbid conditions include: Sickle cell disease, asthma, primary immune deficiency, cardiac disease, chronic pulmonary disease, neurosurgical trauma/procedure, chronic renal insufficiency, immunosuppressive therapy, diabetes mellitus, type I, diabetes mellitus, type II, alcohol abuse, COPD in absence of asthma, rheumatoid arthritis, inflammatory bowel disease, hepatic disease, and long-term corticosteroid use/high-dose steroid use at index date

### Socioeconomic status and the risk of pertussis

The proportions of pertussis cases in the first, second, third, and four quartiles of HOUSES were 28, 33, 31 and 31 %, respectively (*p* = 0.91). Of the 130 pediatric pertussis cases, 102 (78.5 %) had pertussis vaccine up-to-date status whereas 216 of 261 control subjects (82.8 %) had vaccine up-to-date status (*p* = 0.158). Maternal (*p* = 0.40) and paternal (*p* = 0.19) education levels were not associated with the risk of pertussis.

### Socioeconomic status and pertussis vaccine up-to-date status

Out of 55 subjects who missed a pertussis vaccine, 31 missed at least one DTP and 28 at least one DTaP, including 4 who missed both. The pertussis vaccine up-to-date statuses were 63 (79 %), 74 (86 %), 70 (83 %), and 91 (94 %) for children in the first (the lowest SES), second, third, and fourth quartiles (the highest SES) of HOUSES, respectively (Table 2). The odds ratios for having up-to-date pertussis vaccine status in the second, third, and fourth quartiles of HOUSES were 1.82 (95 % CI: 0.79–4.22), 1.22 (95 % CI: 0.54–2.74), and 3.66 (95 % CI: 1.34–9.96), respectively, controlling for age, asthma status prior to index date and comorbidity. The results on multivariate analysis for the association between HOUSES and pertussis vaccine up-to-date status are summarized in Table 2. HOUSES as a continuous variable was positively associated with pertussis vaccine up-to-date statuses adjusting for the same risk factors (adjusted OR: 1.15 per increment of one unit of HOUSES, 95 % CI: 1.04–1.27, *p* = 0.008). Maternal (OR: 0.72, 95 % CI: 0.38–1.35) and paternal (OR:0.49, 95 % CI: 0.22–1.11) education levels were not associated with pertussis vaccine up-to-date status.

**Table 2:** Characteristics of subjects with regard to pertussis vaccine up-to-date status (*N* = 373)

	Pertussis Vaccine Up to date, Yes (*n* = 318)	Pertussis Vaccine Not up to date, No (*n* = 55)	*P*-values*
Age at case index date, years			0.004
Median (IQR)	13.3 (11.5–15.2)	12.5 (7.5–14.2)	
Mean (SD)	12.7 (3.4)	10.7 (4.9)	
Sex, no. (%)			0.78
Male	174 (54.7)	29 (52.7)	
Female	144 (45.3)	26 (47.3)	
Ethnicity, no. (%)			0.47
Hispanic/Latino	3 (0.9)	0 (0)	
American Indian/Alaska Native	2 (0.)	1 (1.8)	
Asian	2 (0.9)	0 (0)	
Native Hawaiian/Pacific Islander	3 (0.6)	1 (1.8)	
White	256 (80.5)	37 (67.3)	
Unknown	52 (16.4)	16 (29.1)	
Ethnicity, no. (%)			0.66
Nonwhites	10 (3.1)	2 (3.6)	
Whites	256 (80.5)	37 (67.3)	
Unknown	52 (16.4)	16 (29.1)	
Maternal educational level, no. (%)			0.38
High school or less	22 (6.9)	2 (3.6)	
Some college	40 (12.6)	7 (12.7)	
College graduate	34 (10.7)	2 (3.6)	
Graduate degree or greater	22 (6.9)	1 (1.8)	
Unknown	200 (62.9)	43 (78.2)	
Paternal education level			0.19
High school or less	11 (3.5)	3 (5.5)	
Some college	31 (9.8)	2 (3.6)	
College graduate	18 (5.7)	1 (1.8)	
Graduate degree or greater	31 (9.8)	1 (1.8)	
Unknown	227 (71.4)	48 (87.3)	
HOUSES index in z-score			<0.001
Median (IQR)	−0.17 (−1.96, 2.51)	−0.93 (−4.05, 0.98)	
Mean (SD)	0.39 (3.59)	−1.23 (2.92)	
HOUSES index in quartiles			0.03
First (lowest SES)	63/298 (21.1)	17/49 (34.7)	
Second	74/298 (24.8)	12/49 (24.5)	
Third	70/298 (23.5)	14/49 (28.6)	
Fourth (highest SES)	91/298 (30.5)	6/49 (12.2)	
Smoking status, no. (%)			0.51
Non-smoking	99 (31.1)	13 (23.6)	
Smoking	45 (14.2)	4 (7.3)	
Unknown	174 (54.7)	38 (69.1)	
Asthma history			
Yes (before index date)	114 (35.8)	11 (20.0)	0.021
No	204 (64.2)	44 (80.0)	
Comorbid conditions at index date, no. (%)^a^			0.63
Yes	7 (2.2)	2 (3.6)	
No	311 (97.8)	53 (96.4)	

*By unconditional logistic regression

^a^Comorbid conditions include: Sickle cell disease, asthma, primary immune deficiency, cardiac disease, chronic pulmonary disease, neurosurgical trauma/procedure, chronic renal insufficiency, immunosuppressive therapy, diabetes mellitus, type I, diabetes mellitus, type II, alcohol abuse, COPD in absence of asthma, rheumatoid arthritis, inflammatory bowel disease, hepatic disease, and long-term corticosteroid use/high-dose steroid use at index date

## Discussion

Our study results showed that conventional SES measures such as parental education levels are not commonly available in medical records, making it difficult to apply them to clinical studies. While HOUSES index is not associated with the risk of pertussis, it predicts pertussis vaccine up-to-date status suggesting HOUSES index might be a useful tool for vaccine delivery research.

Previous studies have shown epidemiologic risk factors for pertussis such as gender [28], vaccination status [28], day care [29–33], age [5, 34], minority ethnic groups, and exposure to tobacco smoke [35]. While vaccination can prevent pertussis infection [36, 37], there is little literature demonstrating reliable factors that predict who gets the vaccine, or describing populations that carry greater percentages of individuals under-vaccinated for the pertussis vaccine. While lower SES is often related to limited access to health care, there have been few studies directly linking SES to pertussis vaccine uptake, and the studies that do exist use SES proxies such as occupation, smoking status, and parental income [38]—variables which we have found to be temporally dynamic and fluctuant, inaccurately recorded in medical records, and frequently absent. For example, in this study dataset, subject ethnicity was 18.7 % unknown and parental education level was 66–75 % unknown. The unavailability of conventional SES measures for clinical research has been a significant barrier to health disparities research, in particular for research on pediatric vaccination.

HOUSES shows promise to overcome the unavailability of conventional SES measures in clinical and administrative datasets and, therein, may enhance a large-scale health disparities research based on administrative or clinical datasets concerning vaccine delivery as shown in our study results. Again, of our 391 pediatric subjects, the addresses for 363 (93 %) subjects were successfully geocoded and formulated for HOUSES index while maternal and paternal education levels were only available for 34 and 25 % of subjects, respectively. As such, maternal and paternal education levels in our study were not associated with pertussis vaccine up to date status. This lack of association might be due to a type 2 error stemming from missing data points in a significantly reduced sample size. In addition, the pattern of missing was not at random. To underscore the point, a large prospective study by Henderson, et al., did show an association between maternal education and pertussis vaccination [39]. Importantly, while maternal education levels did not show any patterns in missing data in relation to SES measured by HOUSES, paternal education levels among children from lower SES background measured by HOUSES were more likely to have missing data on paternal education levels than those from higher SES (data not shown). Therefore, conventional SES measures such as parental education level are frequently unavailable for clinical research and available SES measures might potentially bias the results. In this respect, HOUSES offers a propitious new model for SES measures with superior availability.

In addition, the ease of formulating HOUSES further supports its utility in disparities research concerning vaccine delivery. The weighted kappa index for the agreement between HOUSES and maternal and paternal education levels in quartiles was 0.30 (95 % CI: 0.22–0.37) and 0.38 (95 % CI: 0.25–0.51) suggesting moderate concordance. This result suggests that there is little redundancy in capturing one’s SES measured by HOUSES index compared to other SES measures and HOUSES can be a supplementary SES measure even if other SES measures are available.

Regardless of the merit of HOUSES as compared to other SES metrics, HOUSES was associated with pertussis vaccine up-to-date status with an adjusted OR of 1.15 per increment of one unit of HOUSES. The association between HOUSES index and pertussis vaccine up-to-date status was almost a dose-response relationship as shown in Table 3. The difference in pertussis vaccine up-to-date status between the first and fourth quartiles of HOUSES index was 15 %. When we adjusted the results for potential confounding factors such as comorbidity, asthma status, and even the risk of pertussis (a proxy for unknown risk factors for pertussis), the results were still significant.

**Table 3:** Multivariate analysis for the association between HOUSES Index and pertussis vaccine up-to-date status (*n* = 347)

	Univariate analysis OR (95 % CI), *p*-value	Multivariate analysis OR (95 % CI)^a^
HOUSES in a continuous variable	1.16 (1.05–1.28), *p* = 0.003	1.15 (1.04–1.27)
HOUSES in quartiles		
First (lowest SES)	Referent	Referent
Second	1.66 (0.74–3.74)	1.82 (0.79–4.22)
Third	1.35 (0.62–2.96)	1.22 (0.54–2.74)
Fourth (highest SES)	4.09 (1.52–10.96)	3.66 (1.34–9.96)

^a^Adjusted for age, a history of asthma prior to index date of pertussis and comorbid condition

However, although HOUSES was associated with vaccine up-to-date status, it was not associated with the risk of pertussis. One possible explanation for this finding has to do with the waning efficacy of the pertussis vaccine over time as recently reported. For example, a previous study reported waning humoral immunity in adolescents [7]. Given that the median age of our pediatric subjects was 13.1, it is possible that a large proportion of our adolescent population may have had waned antibody titers after their initial primary series of pertussis vaccine [6]. This could be why current pertussis epidemiology primarily affects adolescents as shown in our community and elsewhere [7].

One important advantage of the HOUSES index over other conventional SES measures is its built-in ability to facilitate a geospatial analysis to identify “hot spots” scaled by vaccination rate (*vaccine topography*). The HOUSES index is linked to a parcel identification number, the unique identification code for a parcel containing one (in the case of single family dwellings) or more residential units. Further studies might also examine the relationship of HOUSES to vaccine up-to-date status. The flu vaccine, for example, with an associated geospatial analysis of “hot spots” in a community where flu transmission is more likely, could facilitate real-time intervention by a public health team by contacting or reminding those without vaccinations, or possibly setting up extra vaccination sites in the geographical location of greatest need as demonstrated by HOUSES.

The strength of our study addresses an apparent need in health research: the paucity of readily available SES measures in commonly used clinical datasets. Our novel application of housing data to assess pertussis vaccination up-to-date status eliminates recall and social desirability bias of prior indices for measuring SES. This study is based on a previous population-based case-control study cohort and has epidemiological advantages as our study population is in a relatively self-contained geographic location and all medical events are linked to each resident and health care provider through the work of the REP program. This increases the likelihood that our dataset includes almost all the cases of pertussis that occurred during the outbreak of 2004–2005.

However, as a retrospective study, there are inherent limitations in validity, reliability, and availability of pertinent data as shown in parental education levels. Furthermore, Olmsted County, Minnesota, is a relatively homogenous population with a small minority population; this too limits the external validity of our findings. Our study only included parental education levels as a reference SES measure as other measures were not available. Furthermore, while HOUSES can explain some aspects of the material deprivation of families, it cannot describe or estimate the deleterious effects of immaterial deprivation (psychosocial, etc.). HOUSES is best understood as a tool to look at the association between illness and the physical qualities of the conditions in which a child lives. Just as other proxies of SES have limitations, so HOUSES does as well.

## Conclusion

In conclusion, our results demonstrate a significant association between socioeconomic status, as measured by HOUSES, and pertussis vaccination up-to-date status. Further studies are necessary to apply HOUSES to the uptake of other vaccines such as the influenza vaccine and to develop strategies for improving vaccine uptake rate through targeting (or hot spotting) underserved populations.

## Abbreviations

HOUSES: HOUsing-based measure of SocioEconomic Status
OR: Odds ratio
SES: Socioeconomic status

## References

[1] CDC. Centers for Disease Control and Prevention, Pertussis. National Immunization Program,0.2005

[2] Novello AC (1994). The role of the public health system in child day-care health. Pediatrics.

[3] Wirsing-von-König CH, Postels-Multan S, Schmitt HJ, Bock HL (1995). Pertussis in adults: frequency of transmission after household exposure. Lancet.

[4] Black S (1997). Epidemiology of pertussis. Pediatr Infect Dis J.

[5] Güriş D, Strebel PM, Bardenheier B, Brennan M, Tachdjian R, Finch E, Wharton M, Livengood JR (1999). Changing epidemiology of pertussis in the United States: increasing reported incidence among adolescents and adults, 1990–1996. Clin Infect Dis.

[6] Klein NP, Bartlett J, Rowhani-Rahbar A, Fireman B, Baxter R (2012). Waning protection after fifth dose of acellular pertussis vaccine in children. N Engl J Med.

[7] Cherry JD, Chang SJ, Klein D, Lee M, Barenkamp S, Bernstein D, Edelman R, Decker MD, Greenberg DP, Keitel W (2004). Prevalence of antibody to Bordetella pertussis antigens in serum specimens obtained from 1793 adolescents and adults.[see comment]. Clin Infect Dis.

[8] Theofiles AG, Cunningham SA, Chia N, Jeraldo PR, Quest DJ, Mandrekar JN, Patel R (2012). Pertussis outbreak, southeastern Minnesota. Mayo Clin Proc.

[9] Higgins MWKJ, Metzner HL (1977). Smoking, socioeconomic status, and chronic respiratory disease. Am Rev Respir Dis.

[10] Nascimento-Carvalho CM, Rocha H, Benguigui Y (2002). Effects of socioeconomic status on presentation with acute lower respiratory tract disease in children in Salvador, Northeast Brazil. Pediatr Pulmonol.

[11] Cohen S (1999). Social status and susceptibility to respiratory infections. Ann N Y Acad Sci.

[12] Majorana A, Cagetti M, Bardellini E, Amadori F, Conti G, Strohmenger L, Campus G (2014). Feeding and smoking habits as cumulative risk factors for early childhood caries in toddlers, after adjustment for several behavioral determinants: a retrospective study. BMC Pediatr.

[13] Krieger N (1992). Overcoming the absence of socioeconomic data in medical records: validation and application of a census-based methodology. Am J Public Health.

[14] Diez-Roux AV, Kiefe CI, Jacobs DR, Haan M, Jackson SA, Nieto FJ, Paton CC, Schulz R (2001). Area characteristics and individual-level socioeconomic position indicators in three population-based epidemiologic studies. Ann Epidemiol.

[15] Hausauer AK, Swetter SM, Cockburn MG, Clarke CA (2011). Increases in melanoma among adolescent girls and young women in california: Trends by socioeconomic status and uv radiation exposure. Arch Dermatol.

[16] Simon AE, Lukacs SL, Mendola P (2011). Emergency department laboratory evaluations of fever without source in children aged 3 to 36 months. Pediatrics.

[17] Lang DM, Polansky M, Sherman MS (2009). Hospitalizations for asthma in an urban population: 1995–1999. Ann Allergy Asthma Immunol.

[18] Kattan M, Mitchell H, Eggleston P, Gergen P, Crain E, Redline S, Weiss K, Evans R, Kaslow R, Kercsmar C (1997). Characteristics of inner-city children with asthma: the National Cooperative Inner-City Asthma Study. Pediatr Pulmonol.

[19] Capili CR, Hettinger A, Rigelman-Hedberg N, Fink L, Boyce T, Lahr B, Juhn YJ (2012). Increased risk of pertussis in patients with asthma. J Allergy Clin Immunol.

[20] Juhn YJ, Beebe TJ, Finnie DM, Sloan J, Wheeler PH, Yawn B, Williams AR (2011). Development and initial testing of a new socioeconomic status measure based on housing data. J Urban Health.

[21] Butterfield MC, Williams AR, Beebe T, Finnie D, Liu H, Liesinger J, Sloan J, Wheeler PH, Yawn B, Juhn YJ (2011). A two-county comparison of the HOUSES index on predicting self-rated health. J Epidemiol Community Health.

[22] Johnson MD, Urm SH, Jung JA, Yun HD, Munitz GE, Tsigrelis C, Baddour LM, Juhn YJ (2012). Housing data-based socioeconomic index and risk of invasive pneumococcal disease: an exploratory study. Epidemiol Infect.

[23] Harris MN LM, Finnie DM, Williams AR, Beebe TJ, Sloan JA, Yawn BY, Juhn YJ. Application of a novel socioeconomic measure using individual housng data in asthma research. Prim Care Respir J. 2014, In press 10.1038/npjpcrm.2014.18 PMC4498187 24965967

[24] Juhn Y, Crowson C, Rand-Weaver J, Krusemark E, Gabriel SE. Application of a novel measure of socioeconomic status using individual housing data in rheumatology research. American College of Rheumatology Annual Meeting 2013, Abstract

[25] Bang DW, Manemann SM, Gerber Y, Roger VL, Lohse CM, Rand-Weaver J, Krusemark E, Yawn BP, Juhn YJ (2014). A novel socioeconomic measure using individual housing data in cardiovascular outcome research. Int J Environ Res Public Health.

[26] Rocca WA, Yawn BP, St Sauver JL, Grossardt BR, Melton LJ (2012). History of the Rochester Epidemiology Project: half a century of medical records linkage in a US population. Mayo Clin Proc.

[27] Broder KR, Cortese MM, Iskander JK, Kretsinger K, Slade BA, Brown KH, Mijalski CM, Tiwari T, Weston EJ, Cohn AC (2006). Preventing tetanus, diphtheria, and pertussis among adolescents: use of tetanus toxoid, reduced diphtheria toxoid and acellular pertussis vaccines recommendations of the Advisory Committee on Immunization Practices (ACIP). MMWR Recomm Rep.

[28] Berger F, Njamkepo E, Minaberry S, Mayet A, Haus-Cheymol R, Verret C, Massit B, Guiso N, Spiegel A (2010). Investigation on a pertussis outbreak in a military school: risk factors and approach to vaccine efficacy. Vaccine.

[29] Richardson M, Elliman D, Maguire H, Simpson J, Nicoll A (2001). Evidence base of incubation periods, periods of infectiousness and exclusion policies for the control of communicable diseases in schools and preschools [see comment] [erratum appears in Pediatr Infect Dis J 2001 Jul;20(7):653]. Pediatr Infect Dis J.

[30] Srugo I, Benilevi D, Madeb R, Shapiro S, Shohat T, Somekh E, Rimmar Y, Gershtein V, Gershtein R, Marva E (2000). Pertussis infection in fully vaccinated children in day-care centers. Israel Emerg Infect Dis.

[31] Christie CD, Marx ML, Daniels JA, Adcock MP (1997). Pertussis containment in schools and day care centers during the Cincinnati epidemic of 1993. Am J Public Health.

[32] Hinman AR (1986). Vaccine-preventable diseases and child day care. Rev Infect Dis.

[33] Anonymous (1984). Public health considerations of infectious diseases in child day care centers. The Child Day Care Infectious Disease Study Group. J Pediatr.

[34] Yunginger JW, Reed CE, O’Connell EJ, Melton LJ, O’Fallon WM, Silverstein MD (1992). A community-based study of the epidemiology of asthma. Incidence rates, 1964–1983. Am Rev Respir Dis.

[35] De Serres G, Duval B (2005). Pertussis vaccination beyond childhood. Lancet.

[36] CDC. Centers for Disease Control and Prevention. Vaccines and immunizations. Pertussis. epidemiology and prevention of vaccine-preventable diseases. The pink book: course textbooks 2011, 12th ed

[37] Broutin H, Viboud C, Grenfell BT, Miller MA, Rohani P (2010). Impact of vaccination and birth rate on the epidemiology of pertussis: a comparative study in 64 countries. Proc R Soc B Biol Sci.

[38] Grant CC, Roberts M, Scragg R, Stewart J, Lennon D, Kivell D, Ford R, Menzies R (2003). Delayed immunisation and risk of pertussis in infants: unmatched case-control study. BMJ.

[39] Henderson J, North K, Griffiths M, Harvey I, Golding J (1999). Pertussis vaccination and wheezing illnesses in young children: prospective cohort study. BMJ.

